# Thermodynamic study of the cerium–cadmium system^☆^

**DOI:** 10.1016/j.calphad.2013.07.005

**Published:** 2014-03

**Authors:** Barbara Skołyszewska-Kühberger, Thomas L. Reichmann, Rajesh Ganesan, Herbert Ipser

**Affiliations:** a Department of Inorganic Chemistry/Materials Chemistry, University of Vienna, A-1090 Wien, Austria; b Indira Gandhi Center for Atomic Research, Kalpakkam, Tamilnadu, India

**Keywords:** Ce–Cd alloys, Ce–Cd: Vapor pressure measurements, Ce–Cd: Thermodynamic properties, Ce–Cd: Phase boundaries

## Abstract

Cadmium vapor pressures were determined over Ce–Cd samples by an isopiestic method. The measurements were carried out in the temperature range from 690 to 1080 K and over a composition range of 48–85 at% Cd. From the vapor pressures thermodynamic activities of Cd were derived for all samples at their respective sample temperatures, and partial molar enthalpies of Cd were obtained from the temperature dependence of the activities. With these partial molar enthalpies the Cd activities were converted to a common temperature of 823 K. By means of a Gibbs–Duhem integration Ce activities were calculated, using a corresponding literature value for the two-phase field (CeCd_11_+L) as integration constant. Finally integral Gibbs energies were calculated for the composition range 48–100 at% Cd with a minimum value of −37 kJ g-atom^−1^ at 823 K in the phase CeCd. Phase boundaries of the intermetallic compounds CeCd, CeCd_2_, Ce_13_Cd_58_, and CeCd_11_ were estimated from the vapor pressure measurements and from SEM analyses.

## 1 Introduction

The nuclear waste disposal is one of the key issues for future use of nuclear energy. Currently several different reprocessing techniques do exist. During traditional aqueous methods some restrictions occur like limited solubility of fuel materials in acidic aqueous solutions and poor radiation stability of the organic solvents employed in the extraction process [1]. The pyrochemical separation techniques seem to be more effective methods for reprocessing of spent high burn-up fuels. The central step in these non-aqueous methods is the electro-refining process where in an electro transportable cell chopped fuel rods are reprocessed [2]. This electro transportable cell consists of a steel anode in form of a basket, where spent fuels are inserted, and two cathodes: a stainless steel cathode for the recovery of U and a liquid metal cathode (using Al [1], Bi [3] or Cd [3]) for the selective recovery of Pu and minor actinides (MA). The entire cell is completely filled with molten LiCl–KCl electrolyte with an additional liquid metal pool occupying its bottom. A variety of liquid metals (Me=Al [1], Bi [3] or Cd [3]) have been explored to extract the rare earth (RE) elements (light rare earth elements between La and Gd with the exception of Pm), which are partially oxidized, from the electrolyte. The extraction behavior is primarily affected by the formation of intermetallic compounds, and thus a thorough knowledge on the existence and stability of intermetallic compounds in the various binary phase diagrams RE-Me are important. Moreover, thermodynamic properties such as the stability of the intermetallic compounds are of high interest, both for a thermodynamic assessment of the binary system based on the CALPHAD^1^ method [4], but also for an optimization of the extraction process itself.

This was the starting point for the present study which wants to provide partial and integral thermodynamic properties of binary Ce–Cd alloys, mainly based on Cd vapor pressure measurements with an isopiestic method [5], [6]. Using limited literature information on partial thermodynamic properties of Ce, integral Gibbs energies of formation could be obtained over a large composition range. Together with calorimetric measurements to determine enthalpies of formation of several intermetallic compounds and a careful experimental phase diagram reinvestigation (both currently under way) this will serve as input for a CALPHAD optimization of the binary Ce–Cd system.

## 2 Literature review

### 2.1 Phase diagram

The Ce–Cd phase diagram appears to be reasonably well known. Johnson et al. [7] determined a partial phase diagram in the Cd-rich part, i.e. they established the liquidus line in the composition range up to about 1 at% Ce by measuring the solubility of Ce in liquid Cd by chemical analysis; they also examined the peritectic decomposition temperature of CeCd_11_ by differential thermal analysis (DTA). Iandelli and Ferro [8] who applied micrography and X-ray diffraction (XRD), reported the existence of the six compounds CeCd, CeCd_2_, CeCd_3_, Ce_2_Cd_9_, CeCd_6_ and CeCd_11_. The complete phase diagram was experimentally investigated by Canepa et al. [9] applying DTA, XRD and metallography. They found one additional high-temperature phase (Ce_2_Cd_17_). Their phase diagram version was the basis for the compilation by Okamoto [10] whereas Massalski et al. [11] still showed only a partial phase diagram. Systematic trends in the phase diagrams of the RE-Cd systems were discussed in a compilation by Gschneidner and Calderwood [12].

According to Okamoto [10] the system is characterized by seven intermetallic compounds, of which three (CeCd, CeCd_2_, and Ce_13_Cd_58_) show a congruent melting behavior and the others (CeCd_3_, CeCd_6_, Ce_2_Cd_17_, and CeCd_11_) are formed through peritectic reactions. All compounds are shown as line compounds without any significant homogeneity range. Only very recently Piao et al. [13] discussed in detail the crystal structure of the compound Ce_13_Cd_58_ and its complicated disorder mechanism.

### 2.2 Thermochemical data

One of the earliest results on this system was given by Elliot and Lemons [14]. They used an isopiestic balance to determine the activity coefficient of Cd over the CeCd_~6_ compound at two selected temperatures: 847 and 912 K. Bayanov and Serebrennikov [15] measured the activity of Ce in dilute solutions of Ce in Cd by a molten salt based emf technique in the temperature range 673–823 K and calculated the excess thermochemical properties. Johnson and Yonco [16] also used a molten salt based emf method to determine the Ce activity in the Cd-rich liquid phase in the temperature range 638–884 K and derived the molar Gibbs energy of formation of CeCd_11_, using the solubility values of Ce in Cd reported by Johnson et al. [7]. Colinet and Pasturel [17] compared the enthalpies and entropies of formation of CeCd_11_measured by Bayanov and Serebrennikov [15] and Johnson and Yonco [16]. More recently Kurata and Sakamura [18] presented a thermodynamic assessment of RE-Cd systems although only limited information was provided for the Ce–Cd system.

## 3 Experimental

**Fig. 1:**
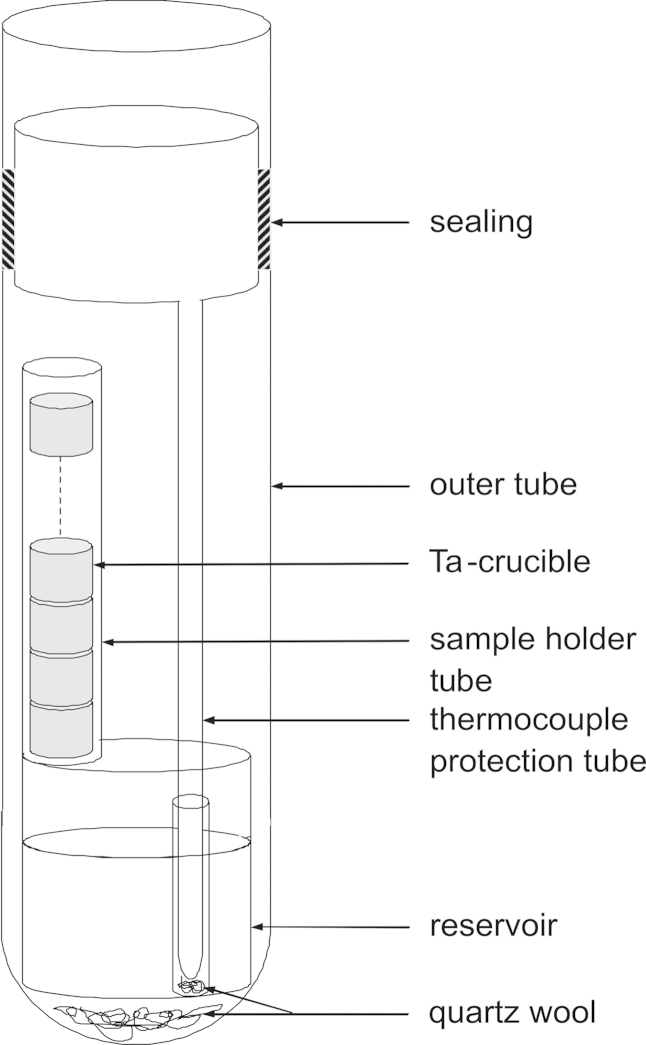
The isopiestic quartz glass apparatus.

The principle and experimental details of the isopiestic method applied in this work were described previously by Ipser et al. [5], [6]. A schematic diagram of the particular setup used in the present investigation is shown in Fig. 1. The apparatus is essentially made of quartz glass. It consists of an outer tube of 38 mm O.D. with one end closed and the other end fitted with a ground joint which can be connected to a vacuum pump. A quartz glass crucible with 32 mm O.D. is placed at the bottom serving as a reservoir for Cd. On top of the reservoir, a quartz glass spacer of suitable height and a quartz supporting tube (15 mm O.D.) are located where the tantalum crucibles containing pure Ce as samples are inserted. An inner tube of 7 mm O.D. with its upper end widened to 32 mm O.D. is used as a thermocouple well. The apparatus can be sealed under vacuum in its upper part.

Before use the entire apparatus was cleaned with an acid mixture (HF/HNO_3_/H_2_O), rinsed with distilled water and dried. Afterwards the fully assembled setup, including the empty tantalum crucibles (approximately 20), was degassed under vacuum (10^−3^ mbar) at 900 °C for 5 h. All preparations for the experiments were then carried out under Ar atmosphere in a glove box. The reservoir was filled with 25 to 35 g of Cd (99.9999% Alfa AESAR, Karlsruhe, Germany), depending on the experimental reservoir temperature. Between 150 and 200 mg of pure Ce (99.9% Alfa AESAR, Karlsruhe, Germany, and smart-elements, Vienna, Austria) were weighed into each Ta crucible with an accuracy of ±0.1 mg. The assembled apparatus was brought outside the glove box securely closed using a glass stopper. It was connected to the vacuum pump, evacuated and sealed under a dynamic vacuum of better than 10^−3^ mbar.

The isopiestic equilibration experiments were carried out in different temperature gradients, applied by two-zone furnaces, for periods of about 3 to 5 weeks. The temperatures of the samples (*T*_S_) and the reservoir (*T*_R_) were measured periodically by raising a Pt/Pt10%Rh thermocouple inside the thermocouple well. After equilibration, the isopiestic apparatus was quenched in cold water and cut open by a diamond saw. The individual samples (which had become Ce–Cd alloys during the equilibration) were weighed again and their compositions were derived from the mass difference which was attributed to the uptake of Cd.

Representative samples were characterized by XRD with Cu *K*α radiation on a Bruker D8 Advance Diffractometer with Bragg–Brentano geometry. The XRD patterns were analyzed and refined by means of the TOPAS 3 software (provided by Bruker), applying the fundamental parameter approach for peak profile modeling. In order to check the calculated compositions selected samples were analyzed by scanning electron microscopy (SEM; Zeiss Supra 55 VP) using energy dispersive X-ray spectroscopy (EDS).

## 4 Results and discussion

### 4.1 Isopiestic measurements

Eight successful isopiestic experiments were carried out for the Ce–Cd system, with reservoir temperatures between 673 and 851 K corresponding to total vapor pressures of Cd between about 2 and 76 mbar, respectively. The corresponding sample temperatures were between 690 and 1080 K. Since the vapor pressure of Ce is several orders of magnitude lower compared to that of Cd it can be neglected, and it can be assumed that the total pressure in the system is due to Cd maintained at a constant temperature in the reservoir. When the final equilibrium is reached in an isopiestic experiment the partial pressure of Cd over each sample at its sample temperature *T*_S_, *p*_Cd_(*T*_S_), is equal to the vapor pressure of pure Cd at the reservoir temperature *T*_R_, *p*^0^_Cd_(*T*_R_):

(1) $$p_{\mathrm{Cd}}(T_{S})=p_{\mathrm{Cd}}^{0}(T_{R})$$

Under these circumstances the Cd activity in the samples can be calculated by the following equation:

(2) $$a_{\mathrm{Cd}}(T_{S})=\frac{p_{\mathrm{Cd}}(T_{S})}{p_{\mathrm{Cd}}^{0}(T_{S})}=\frac{p_{\mathrm{Cd}}^{0}(T_{R})}{p_{\mathrm{Cd}}^{0}(T_{S})}$$

The vapor pressure of pure Cd as a function of temperature was taken from Binnewies and Milke [19]:

(3) $$\log\left(\frac{p_{\mathrm{Cd}}^{0}}{\mathrm{bar}}\right)=8.7-5690\times \frac{K}{T}-1.07\times \log\frac{T}{K}$$

**Fig. 2:**
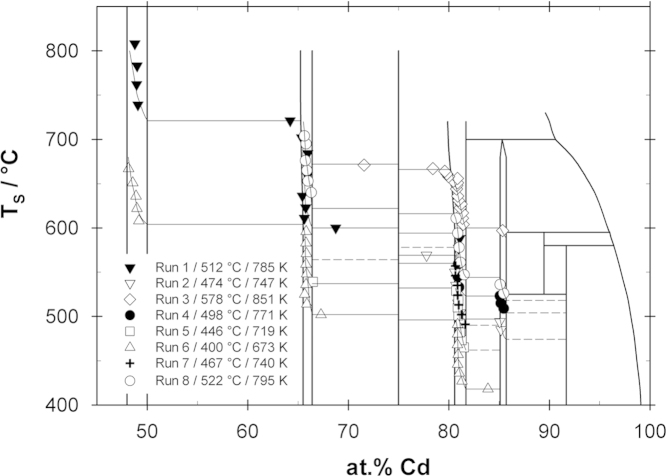
Sample temperature vs. sample composition superimposed on the partial Ce–Cd phase diagram (dashed lines in two-phase fields are estimated and not supported directly by data points).

The experimental results, i.e. sample temperature, sample composition and thermodynamic activity of Cd for each sample, are listed in Table 1. In Fig. 2 sample temperatures are plotted against sample compositions for all experimental runs (the so-called equilibrium curves) and superimposed on the phase diagram. In order to check the compositions calculated from the weight change, selected samples were analyzed by scanning electron microscopy using energy dispersive X-ray spectroscopy (EDS). In general, the compositions agreed within 0.5 at% and the temperatures are assumed to be accurate within ±2 K. As can be seen from Fig. 2, the equilibrium samples obtained in the experimental runs cover the concentration range between about 48 and 85 at% Cd. The majority of the samples were single phase, namely CeCd, CeCd_2_, Ce_13_Cd_58_ and CeCd_6_. Interestingly, single phase samples of CeCd_3_ could not be obtained in any of the runs suggesting that CeCd_3_ is only slightly more stable than a two-phase mixture of its neighboring compounds. Thus the activities of Cd in the adjacent two-phase fields CeCd_2_+CeCd_3_ and CeCd_3_+Ce_13_Cd_58_ are only slightly different (cf. Fig. 7). Moreover it was found that a majority of data points fall into the composition range of the phase Ce_13_Cd_58_indicating that this must be one of the most stable compounds in the Ce–Cd system, in agreement with its congruent formation from the liquid [10].

**Table 1:** Isopiestic experimental results; standard state: Cd (liquid).

Sample no.	Cd (at%)	*T*_S_ (K)	ln *a*_Cd_ (*T*_S_)	Phases	$\Delta {\overset{¯}{H}}_{\mathrm{Cd}}$ (kJ g-atom^−1^)	ln*a*_Cd_ (823 K)
*Run 1*	T_R_: 785 K. 37 days					
1	81.0	850	−1.19	Ce_13_Cd_58_	−8.5	−1.23
2	81.1	861	−1.37	Ce_13_Cd_58_	−5.1	−1.41
3	68.7	873	−1.56	CeCd_3_+CeCd_2_	−30.3	−1.82
4	65.6	884	−1.75	CeCd_2_	−48.3	−2.24
5	65.8	896	−1.93	CeCd_2_	−45.3	−2.47
6	65.4	909	−2.12	CeCd_2_	−53.0	−2.85
7	65.9	933	−2.46	CeCd_2_	−41.5	−3.17
8	66.0	958	−2.79	CeCd_2_	−40.3	−3.62
9	65.3	976	−3.03	CeCd_2_+CeCd	−53.5	−4.25
10	64.2	994	−3.26	CeCd_2_+CeCd	−53.5	−4.60
11	49.1	1012	−3.47	CeCd	−57.6	−5.04
12	48.9	1036	−3.74	CeCd	−57.9	−5.48
13	49.0	1056	−3.97	CeCd	−57.8	−5.83
14	48.8	1081	−4.23	CeCd	−58.3	−6.27

*Run 2*	*T*_R_*: 747* *K. 42 days*					
1	85.2	758	−0.24	CeCd_6_	−3.1	−0.20
2	85.1	767	−0.43	CeCd_6_	−3.1	−0.40
3	81.1	777	−0.64	Ce_13_Cd_58_	−4.4	−0.60
4	80.9	788	−0.86	Ce_13_Cd_58_	−11.8	−0.78
5	80.7	808	−1.24	Ce_13_Cd_58_	−23.9	−1.18
6	80.6	823	−1.52	Ce_13_Cd_58_	−35.6	−1.52
7	77.8	842	−1.85	Ce_13_Cd_58_+CeCd_3_	−29.8	−1.95

*Run 3*	*T*_R_*: 851* *K. 29 days*					
1	85.4	870	−0.31	CeCd_6_	−3.1	−0.34
2	81.4	877	−0.42	Ce_13_Cd_58_	−2.8	−0.45
3	81.4	884	−0.53	Ce_13_Cd_58_	−2.6	−0.56
4	81.4	890	−0.63	Ce_13_Cd_58_	−2.6	−0.66
5	81.1	896	−0.72	Ce_13_Cd_58_	−4.1	−0.77
6	81.2	900	−0.78	Ce_13_Cd_58_	−3.1	−0.82
7	81.0	904	−0.84	Ce_13_Cd_58_	−8.6	−0.95
8	80.9	909	−0.91	Ce_13_Cd_58_	−12.1	−1.08
9	80.8	917	−1.03	Ce_13_Cd_58_	−19.3	−1.32
10	80.8	921	−1.09	Ce_13_Cd_58_	−17.9	−1.36
11	80.9	924	−1.13	Ce_13_Cd_58_	−13.1	−1.34
12	80.9	929	−1.20	Ce_13_Cd_58_	−13.7	−1.43
13	80.0	931	−1.23	Ce_13_Cd_58_+CeCd_3_	−29.8	−1.73
14	79.8	934	−1.27	Ce_13_Cd_58_+CeCd_3_	−29.8	−1.79
15	79.6	937	−1.31	Ce_13_Cd_58_+CeCd_3_	−29.8	−1.84
16	78.4	940	−1.35	Ce_13_Cd_58_+CeCd_3_	−29.8	−1.89
17	71.6	944	−1.41	CeCd_3_+CeCd_2_	−30.3	−1.97

*Run 4*	*T*_R_*: 771* *K. 32 days*					
1	85.5	782	−0.22	CeCd_6_	−3.1	−0.20
2	85.2	788	−0.34	CeCd_6_	−3.1	−0.32
3	85.1	796	−0.50	CeCd_6_	−3.1	−0.48
4	81.0	806	−0.69	Ce_13_Cd_58_	−7.2	−0.67
5	80.9	818	−0.91	Ce_13_Cd_58_	−11.6	−0.90
6	80.9	832	−1.16	Ce_13_Cd_58_	−10.7	−1.18

*Run 5*	*T*_R_*: 719* *K. 25 days*					
1	81.5	738	−0.44	Ce_13_Cd_58_	−3.4	−0.38
2	81.3	746	−0.62	Ce_13_Cd_58_	−2.7	−0.58
3	81.1	755	−0.82	Ce_13_Cd_58_	−4.1	−0.76
4	81.1	765	−1.03	Ce_13_Cd_58_	−4.8	−0.98
5	81.0	774	−1.22	Ce_13_Cd_58_	−9.0	−1.13
6	80.9	783	−1.40	Ce_13_Cd_58_	−12.6	−1.30
7	81.0	792	−1.58	Ce_13_Cd_58_	−9.0	−1.52
8	80.7	802	−1.77	Ce_13_Cd_58_	−22.3	−1.68
9	66.5	812	−1.96	CeCd_3_+CeCd_2_	−30.3	−1.90

*Run 6*	*T*_R_*: 673* *K. 25 days*					
1	83.9	691	−0.48	CeCd_6_ +Ce_13_Cd_58_	−3.1	−0.39
2	81.3	700	−0.71	Ce_13_Cd_58_	−2.6	−0.64
3	81.0	709	−0.93	Ce_13_Cd_58_	−6.3	−0.78
4	80.9	719	−1.17	Ce_13_Cd_58_	−13.1	−0.90
5	80.9	730	−1.43	Ce_13_Cd_58_	−10.7	−1.23
6	80.9	741	−1.68	Ce_13_Cd_58_	−14.2	−1.45
7	80.9	753	−1.95	Ce_13_Cd_58_	−13.1	−1.77
8	80.9	764	−2.18	Ce_13_Cd_58_	−13.1	−2.03
9	67.3	775	−2.41	CeCd_3_+CeCd_2_	−30.3	−2.14
10	65.9	787	−2.65	CeCd_2_	−42.6	−2.37
11	65.7	798	−2.87	CeCd_2_	−47.5	−2.65
12	65.8	809	−3.08	CeCd_2_	−44.5	−2.96
13	65.9	821	−3.30	CeCd_2_	−42.9	−3.28
14	65.8	832	−3.49	CeCd_2_	−44.5	−3.56
15	65.8	844	−3.70	CeCd_2_	−43.6	−3.86
16	65.8	856	−3.90	CeCd_2_	−44.7	−4.16
17	65.8	869	−4.12	CeCd_2_	−44.2	−4.46
18	49.2	881	−4.31	CeCd	−57.4	−4.86
19	49.0	895	−4.52	CeCd	−57.8	−5.20
20	48.9	909	−4.73	CeCd	−58.0	−5.54
21	48.6	924	−4.95	CeCd	−58.8	−5.89
22	48.1	940	−5.17	CeCd	−59.7	−6.26

*Run 7*	*T*_R_*: 740* *K. 25 days*					
1	81.6	764	−0.52	Ce_13_Cd_58_	−4.5	−0.47
2	81.3	775	−0.75	Ce_13_Cd_58_	−2.7	−0.73
3	81.0	786	−0.97	Ce_13_Cd_58_	−7.9	−0.92
4	80.9	797	−1.19	Ce_13_Cd_58_	−12.6	−1.13
5	80.9	808	−1.40	Ce_13_Cd_58_	−13.1	−1.36
6	80.7	819	−1.60	Ce_13_Cd_58_	−28.3	−1.58
7	80.7	830	−1.80	Ce_13_Cd_58_	−27.4	−1.83

*Run 8*	*T*_R_*: 795* *K. 21 days*					
1	85.5	799	−0.08	CeCd_6_	−3.1	−0.06
2	85.1	809	−0.27	CeCd_6_	−3.1	−0.26
3	81.5	820	−0.47	Ce_13_Cd_58_	−3.5	−0.47
4	81.2	834	−0.72	Ce_13_Cd_58_	−3.9	−0.73
5	81.0	851	−1.01	Ce_13_Cd_58_	−6.9	−1.04
6	80.9	867	−1.28	Ce_13_Cd_58_	−12.1	−1.37
7	80.7	884	−1.55	Ce_13_Cd_58_	−22.3	−1.77
8	66.3	913	−1.98	CeCd_2_	−32.2	−2.49
9	66.0	926	−2.17	CeCd_2_	−40.1	−2.92
10	65.9	938	−2.34	CeCd_2_	−42.4	−3.19
11	65.7	949	−2.48	CeCd_2_	−46.1	−3.44
12	65.8	968	−2.73	CeCd_2_	−44.2	−3.70
13	65.6	977	−2.85	CeCd_2_	−48.2	−3.96

Some of the samples were obtained in various two-phase fields after equilibration, i.e. CeCd_6_+Ce_13_Cd_58_, Ce_13_Cd_58_+CeCd_3_, CeCd_3_+CeCd_2_ and CeCd_2_+CeCd. This was probably caused by slight variations in the sample temperatures. SEM investigations of some respective two-phase samples were used to define phase boundaries and homogeneity ranges of the compounds CeCd_6_, Ce_13_Cd_58_, CeCd_2_ and CeCd (see Table 2).

**Table 2:** Phase boundaries at 823 K from isopiestic vapor pressure measurements (Fig. 2) and from SEM analyses.

Phase	Phase boundaries (at% Cd)	
	From isopiestic	From SEM
CeCd	48.0–50.0	48.0–50.9
CeCd_2_	65.4–66.4	65.5–66.4
Ce_13_Cd_58_	80.6–81.7	80.6–81.7
CeCd_6_	85.1–85.7	85.8–86.3

### 4.2 Partial enthalpy of mixing of Cd

**Fig. 3:**
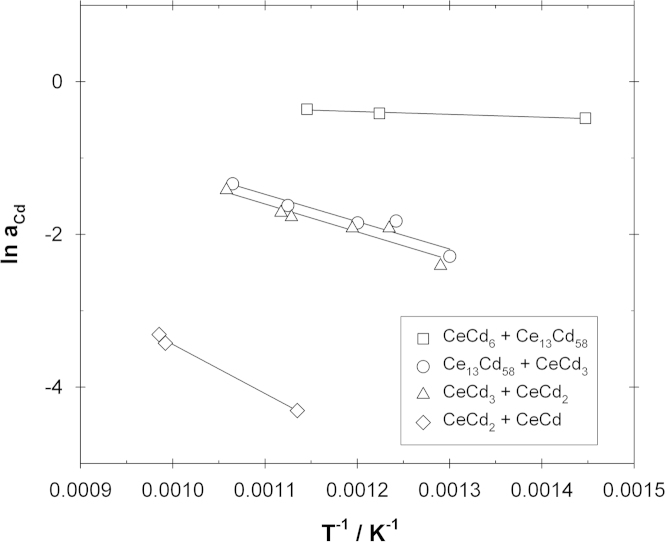
Natural logarithm of the Cd activity vs. reciprocal temperature in four two-phase fields.

From the Cd vapor pressures measured over the samples in the four two-phase fields (see above) the corresponding activity values were calculated. They are plotted as ln *a*_Cd_ versus reciprocal temperature in Fig. 3. Assuming straight phase boundaries, i.e. no variation of the solubilities with temperature for the different compounds, one can apply an adapted Gibbs–Helmholtz equation to estimate partial molar enthalpies of mixing of Cd in these two-phase fields:

(4) $$\frac{\partial \ln\;a_{\mathrm{Cd}}}{\partial (1/T)}=\frac{\Delta {\bar{H}}_{\mathrm{Cd}}}{R}$$

Although it is obvious that the phase boundaries are not temperature independent in the investigated temperature range this procedure gives at least a good estimate of the corresponding $\Delta {\bar{H}}_{\mathrm{Cd}}$ values which are also included in Table 1. By comparing the slopes in Fig. 3 it can be concluded that with decreasing Cd concentration the partial molar enthalpy of mixing of Cd becomes more negative. This indicates a clearly exothermic behavior in the Ce–Cd system in the investigated composition range.

**Fig. 4:**
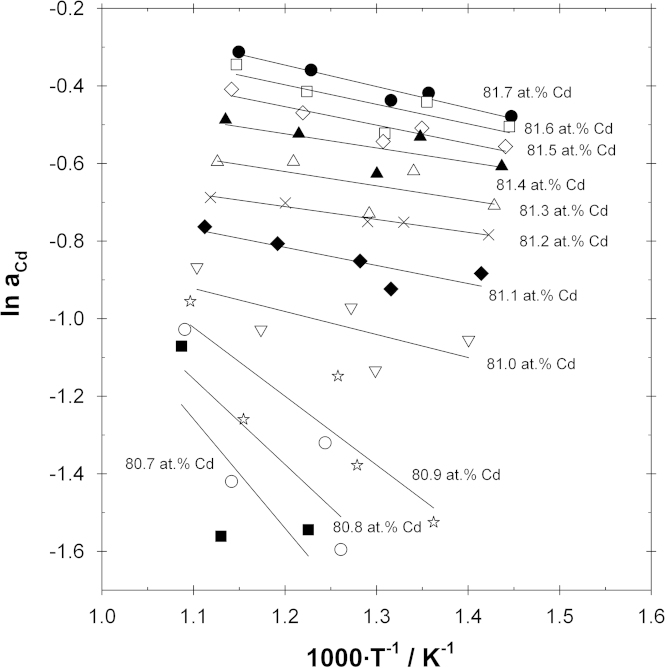
Natural logarithm of the Cd activity vs. reciprocal temperature for selected compositions in the Ce_13_Cd_58_ phase.

**Fig. 5:**
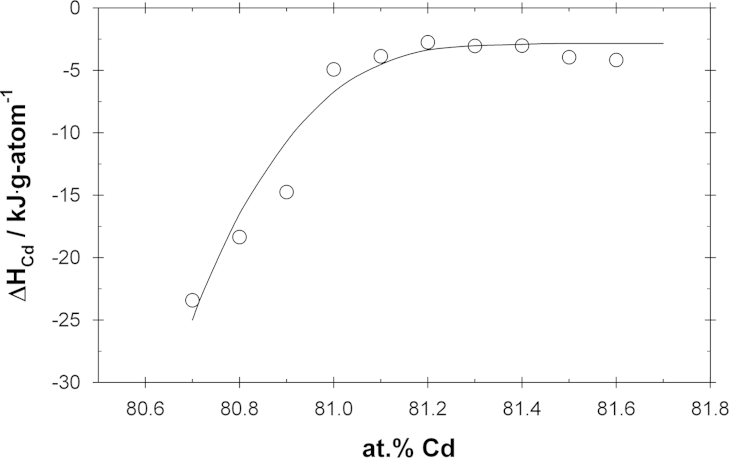
Partial molar enthalpy of cadmium in Ce_13_Cd_58_ along the homogeneity range; standard state: Cd (l).

To derive partial molar enthalpies of mixing of Cd in the phase Ce_13_Cd_58_, sample temperatures for selected compositions were obtained by interpolation from the equilibrium curves in Fig. 2; the activities for these hypothetical samples were calculated according to Eq. (2), and they were plotted as a function of the reciprocal temperature (see Fig. 4). Straight lines were obtained for the selected compositions by linear regression, corresponding to the Gibbs–Helmholtz equation [4]. The calculated partial molar enthalpy values of Cd over the entire homogeneity range of Ce_13_Cd_58_are shown in Fig. 5. The estimated error in the partial enthalpy values is in the range between ±1 and ±3 kJ g-atom^−1^. As can be seen the values vary from close to zero at the Cd-rich end to rather negative values at the Ce-rich end of the homogeneity range, indicating once more that Ce_13_Cd_58_ is a relatively stable phase. An analogous procedure was applied to estimate $\Delta {\bar{H}}_{\mathrm{Cd}}$ values for the phase CeCd_2_ whereas not enough data points were available to allow such an evaluation for the two other phases CeCd_6_and CeCd. Therefore it was assumed that $\Delta {\bar{H}}_{\mathrm{Cd}}$ in CeCd varies linearly between the corresponding values in the neighboring two-phase fields whereas a value of −3.1 kJ g-atom^−1^ was assumed for the Ce-rich end of CeCd_6_ (the same value as in the neighboring two-phase field CeCd_6_+Ce_13_Cd_58_). All partial enthalpy values are included in Table 1.

### 4.3 Thermodynamic activity of Cd

The partial enthalpy values in Fig. 5 (and Table 1) were used to convert the Cd activities in Ce_13_Cd_58_ to a common temperature of 823 K (which represents an average of all experimental sample temperatures) applying an integrated form of the Gibbs–Helmholtz equation:

(5) $$\ln\;a_{\mathrm{Cd}}(T_{2})-\ln\;a_{\mathrm{Cd}}(T_{1})=\frac{\Delta {\bar{H}}_{\mathrm{Cd}}}{R}\times \left(\frac{1}{T_{2}}-\frac{1}{T_{1}}\right)$$

**Fig. 6:**
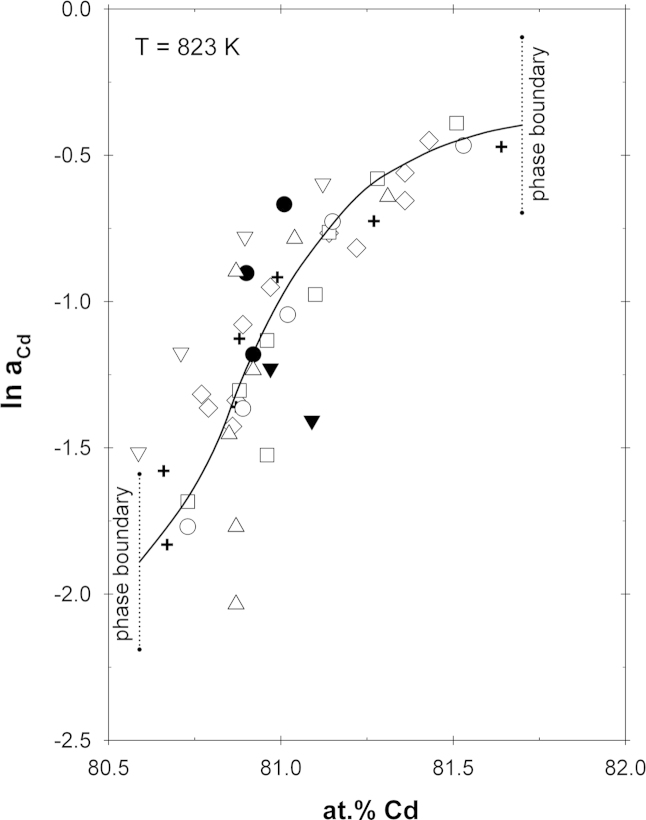
Natural logarithm of the Cd activity in the Ce_13_Cd_58_ phase at 823 K; standard state: Cd(l); symbols equal to Fig. 2.

**Fig. 7:**
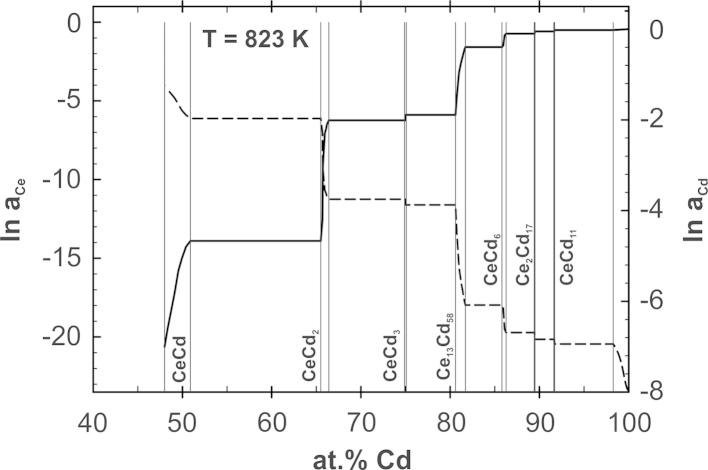
Natural logarithm of the cadmium and cerium activities vs. composition at 823 K.

The normalized activity values are shown in Fig. 6. Once more the drastic change of the Cd activities indicates a relatively high stability of Ce_13_Cd_58_. Using the partial enthalpy values from Table 1, the activities in all other compounds as well as in all the two-phase fields were converted to the same common temperature of 823 K. The uncertainty of the ln *a*_Cd_ (823 K) values is less than 10%. All these values are listed in Table 1, a corresponding plot of ln *a*_Cd_ over the entire composition range between 40 and 100 at% Cd is shown in Fig. 7.

Elliott and Lemons determined Cd activities in the phase CeCd_6_ also by means of an isopiestic method. According to their results ln *a*_Cd_ in this phase varies between −0.458 and −0.119 at 847 K and between −0.343 and −0.098 at 912 K. If the present results are converted to the same temperatures one obtains values between about −0.47 and −0.05 at 847 K and between about −0.44 and −0.02 at 912 K. Obviously, the agreement is quite good.

### 4.4 Activity of Ce and integral Gibbs energy

The Ce activity in the two-phase field CeCd_11_+L was determined by Johnson and Yonco [16] in the temperature range 638–884 K. They calculated partial excess Gibbs energies of Ce based on data for the (CeCd_11_+L)/L phase boundary from an earlier investigation of the Cd-rich part of the Ce–Cd phase diagram by Johnson et al. [7], and presented the following equation:

(6) $$\Delta {\overset{¯}{G}}_{\mathrm{Ce}}^{\mathrm{xs}}=x_{\mathrm{Cd}}^{2}(-179,950+79.20\times T-95,600\times x_{\mathrm{Ce}})$$

with *T* in K and $\Delta {\overset{¯}{G}}_{\mathrm{Ce}}^{\mathrm{xs}}$ in J mol^−1^. With the Ce solubility in liquid Cd and their measured Ce activities Johnson and Yonco used the Gibbs–Duhem relationship to derive Cd activities as well as integral Gibbs energies of formation in the corresponding temperature range. From this they obtained the integral Gibbs energy of formation for the compound CeCd_11_ at 823 K, $\Delta_{f}G({\mathrm{CeCd}}_{11})$=−11.8 kJ g-atom^−1^.

**Fig. 8:**
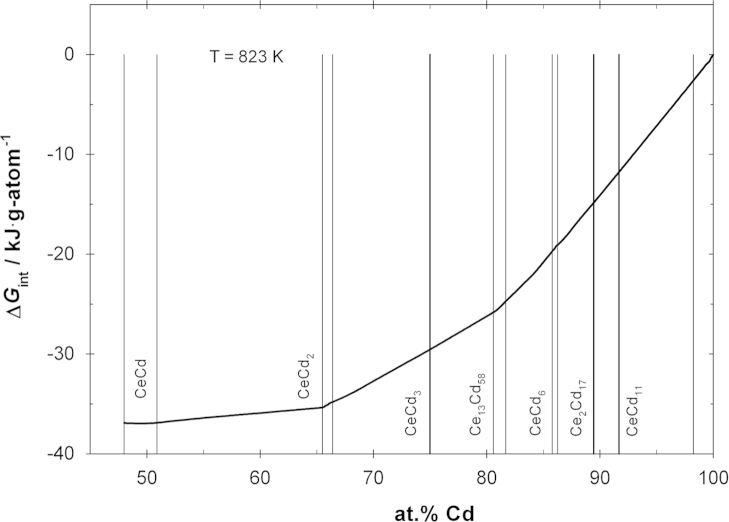
Integral Gibbs energy of formation vs. composition at 823 K; standard states: Ce(s) and Cd(l).

From Eq. (6) ln *a*_Ce_=−20.45 was calculated for the boundary of the liquid phase at 823 K, and this was applied as an integration constant for a Gibbs–Duhem integration based on the present data of ln *a*_Cd_. Thermodynamic activities of Ce were calculated in the composition range 48–85 at% Cd using the so-called α-function as described by Darken and Gurry [20], and are included in Fig. 7. Having activity values both for Ce and Cd, the integral Gibbs energy of formation could be calculated at 823 K for the same composition range. It is shown as a function of composition in Fig. 8.

### 4.5 Homogeneity ranges of intermetallic compounds

As can be seen from Fig. 2, the kinks in the so-called equilibrium curves provide some information on the phase boundaries of some of the intermetallic compounds. It is striking that the homogeneity ranges of CeCd_6,_ Ce_13_Cd_58_, CeCd_2_, and CeCd appear to be shifted to the Ce-rich side of the nominal stoichiometry. For example, the ln *a*_Cd_-curve in Ce_13_Cd_58_ shows an inflection point at about 80.9 at% Cd (cf. Fig. 6) although it would be expected at about 81.7 at% Cd according to its 13/58 stoichiometry. Considering the stoichiometry of Ce_12.60_Cd_58.68_, derived from the crystallographic study by Piao et al. [13], the order composition would be expected at even higher Cd contents, i.e. at about 82.3 at%.

Table 2 shows a comparison of the phase boundaries estimated from the isopiestic vapor pressure measurements (Fig. 2) and those derived from the SEM analyses. As can be seen, the agreement is very good for the phases CeCd_2_ and Ce_13_Cd_58_ whereas there are some discrepancies for CeCd and CeCd_6_. Since the number of isopiestic data points in the latter two phases is rather limited the phase boundaries from SEM should be considered being more reliable. However, this leaves still the question why the homogeneity ranges appear in general shifted to the Ce-rich side. The reason for this is not clear at the moment but may have to do with the actual crystal structures.

## 5 Summary

Thermodynamic activities of Cd were determined for the Ce–Cd system in the temperature range between 690 and 1080 K and the composition range between 48 and 85 at% Cd based on an isopiestic vapor pressure method. The activity values were converted to a common temperature of 823 K and are given for the composition range 48–100 at% Cd. Using a literature value of ln *a*_Ce_ as integration constant, it was possible to calculate Ce activities and integral Gibbs energies of formation for the same temperature and composition range. A minimum of Δ*G*_Cd_=−37 kJ g-atom^−1^ was obtained in the phase CeCd.

The results of the isopiestic measurements were used to obtain phase boundaries for the four intermetallic phases CeCd, CeCd_2_, Ce_13_Cd_58_, and CeCd_6_. These are compared with phase boundaries obtained from SEM measurements, and the agreement is found to be good for CeCd_2_ and Ce_13_Cd_58_, whereas some discrepancies are found for the other two compounds.

## Footnotes

1 CALPHAD=CALculation of PHAse Diagrams.
