# Supplementary material for: Thermodynamic study of the cerium–cadmium system
Source: CALPHAD. 2014 Mar;44:14–20. doi: 10.1016/j.calphad.2013.07.005 (PMC4270483; doi:10.1016/j.calphad.2013.07.005)
Supplement: Supplementary file 1 — Supplementary data [file mmc1.docx]

No phase diagram data is involved.

Regards,

Barbara Skolyszewska-Kühberger
